# QMMAC: Quorum-Based Multichannel MAC Protocol for Wireless Sensor Networks

**DOI:** 10.3390/s21113789

**Published:** 2021-05-30

**Authors:** Eman Alzahrani, Fatma Bouabdallah

**Affiliations:** Information Technology Department, Faculty of Computing and Information Technology, King Abdulaziz University, Jeddah 21589, Saudi Arabia; fothman1@kau.edu.sa

**Keywords:** wireless sensor networks, MAC protocol, quorum system, multichannel communication

## Abstract

In wireless sensor networks, energy conservation is a critical task. Thus, it is crucial to design an effective MAC protocol that minimizes energy consumption while guaranteeing high network throughput and low delay. In this article, we propose a quorum-based multichannel MAC protocol (QMMAC) for corona-based WSNs. QMMAC utilizes the multichannel communication feature and the quorum concept to greatly increase the throughput while conserving energy. The aim of this protocol is to allow each node and all its forwarders to wake up at the same time while avoiding collision and overhearing by separating their simultaneous communications using the multichannel feature. More precisely, the main idea of QMMAC is twofold. First, QMMAC wakeup scheduling is designed to minimize the end-to-end delay by allowing nodes to wake up at exactly the same time as their potential forwarders, whereas nodes that are not acting as forwarders for each other wake up at a completely different time, and thus overhearing, idle listening and collisions are avoided. Second, channel assignment, which also uses the concept of quorums, is used to share data channels so that there is no conflict or additional packet exchange required to negotiate the availability of channels. Thus, the end-to-end delay is further minimized as well as collisions between conflicting neighbors are avoided. Simulation results indicate that the network performance is improved by QMMAC in terms of energy efficiency, throughput and end-to-end delay.

## 1. Introduction

Wireless sensor networks (WSNs) are composed of a vast number of sensors that can sense the environment, collect data, store it and send it for further processing. Sensor nodes are typically battery-powered and scattered in a difficult-to-reach area, which makes replacement or recharging nodes’ batteries impracticable. Therefore, energy conservation is a critical task due to these energy limitations. The main sources of energy consumption in WSNs are (1) transmission, (2) reception, (3) collision, (4) overhearing, and (5) idle listening [1,2]. Energy consumption during data reception and transmission is required for a node to fulfill its task. However, energy consumption during collisions, idle listening, and overhearing are the main sources of energy wastage. In such energy-constrained networks, this energy wastage cannot be tolerated and has to be reduced to the maximum possible extent. Consequently, to minimize energy wastage, developing an energy-efficient MAC protocol for WSNs is crucial, as the MAC protocol is responsible for controlling nodes’ access to the shared wireless medium.

**Figure 1:**
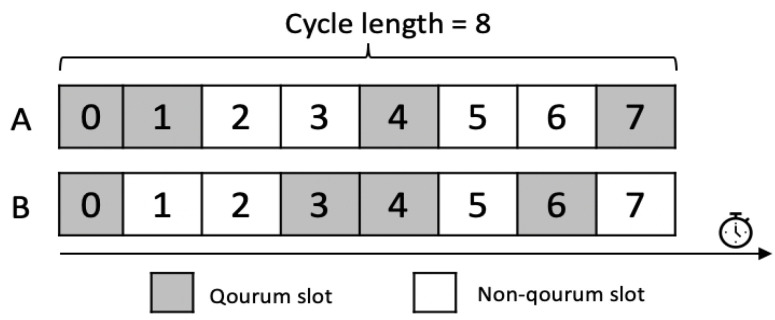
Example of intersecting quorums.

One of the most efficient solutions to improve the energy efficiency and hence prolonging the network lifetime is using the quorum concept to reduce the nodes’ wakeup times. Several quorum-based MAC protocols [3,4,5,6,7,8,9,10,11,12,13,14,15,16] are proposed. The time in these protocols is partitioned into cycles. Each cycle is composed of fixed-size time slots. Each slot in the cycle can be either: quorum slot or non-quorum slot. At the quorum slot, the node wakes up for data transmission and reception. At the non-quorum time slot, the node enters into sleep mode for energy saving. A quorum system is constructed from a universal set that contains all slot numbers within the cycle. Each quorum in the system is a subset of that universal set. A quorum simply represents the slots during which the node is in a wakeup state for potential communication. Then, each node is assigned a quorum set from the quorum system as its wakeup schedule. For instance, node A in Figure 1 is assigned the quorum ${\;Q}_{A}=\left\{0,1,4,7\right\}$ which means that node A will wake up in slots 0, 1, 4 and 7. To allow communication, the quorum system must guarantee that the quorum sets that are assigned to any two neighbor nodes have a number of intersecting wakeup slots. For example, as shown in Figure 1, two nodes A and B, each is assigned a set as its wakeup schedule. Accordingly, $\;Q_{A}=\left\{0,1,4,7\right\}$ and $\;Q_{B}=\left\{0,3,4,6\right\}$ under the universal set  U={0,1,…,8} where the intersection during which A and B can communicate is $\;Q_{A}\;\cap^{\;}\;Q_{B}=\left\{0,4\right\}$.

**Figure 2:**
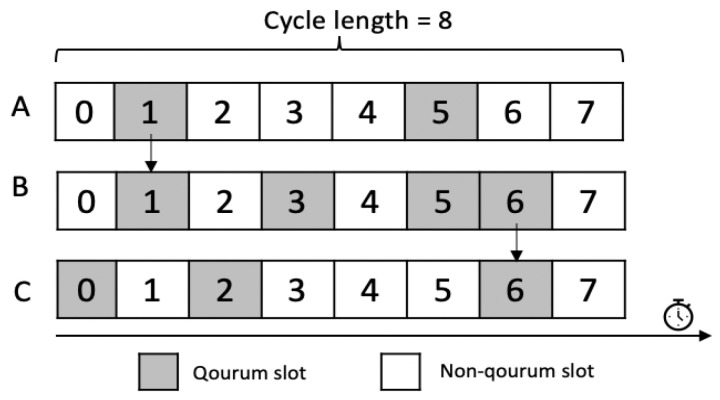
End-to-end delay in quorum-based scheduling.

Note that in this way not only idle listening is avoided but also collision and overhearing. Indeed, thanks to the quorum concept, all nodes in the same neighborhood will get different intersections with each other ($Q_{A}\cap^{\;}Q_{B\;}\neq \;Q_{A}\cap^{\;}\;Q_{C\;}\neq \;Q_{C}\cap^{\;}Q_{B}$), and hence they will not wake up all at the same time slots. Thus, they can communicate with each other without neither collision nor overhearing. In energy-constrained WSNs, quorum-based protocols are clearly a promising solution. It is absolutely true that if appropriate quorum sets are designed, overhearing, collision and idle listening can be avoided; however, the end-to-end delay may increase. In fact, if a node has a data packet, it must buffer this data, then wait for the intersecting wakeup slot with its receiver. For instance, as shown in Figure 2, node A may send a packet to node B in slot 1. If node B wants to forward this packet to node C, it will wait until the next intersecting quorum slot, which is slot 6 in this example. Thus, node B has to wait for four slots in order to be able to forward the message which will highly increase the end-to-end delay.

Another promising solution is utilizing multichannel communication to avoid collisions and overhearing while it reduces the end-to-end delay. By allowing conflicting neighbors to have multiple simultaneous successful transmissions on different channels, this solution is meant to considerably reduce the end-to-end delay. Typically, using a single channel, neighbor nodes cannot communicate simultaneously as collisions will unavoidably occur. However, they can communicate with their intended receivers simultaneously if they are allocated different channels. Therefore, energy consumption and end-to-end delay are expected to be highly decreased as successful packet transmissions can be completed on different channels.

This article proposes a quorum-based multichannel MAC protocol, QMMAC, that takes advantage of both multichannel and quorum concepts to highly increase the throughput while conserving energy. Specifically, QMMAC allows neighbors to wake up at the same time while separating them by the multichannel feature. Accordingly, this protocol is supposed to decrease energy consumption due to idle listening, overhearing, and collision. First, by utilizing the quorum concept, we propose a quorum-based wakeup scheduling that utilizes the line quorum system to decrease the end-to-end delay by allowing nodes to wake up at exactly the same time as their potential forwarders. Accordingly, the overhearing, idle listening and collisions are avoided as nodes that are not forwarders to each other will wake up at a completely different time. However, overhearing and collisions may occur between wakeup neighbor nodes. To avoid collision and overhearing between these neighbor forwarders, a multichannel quorum-based solution is used. According to QMMAC, every node with each one of its potential forwarders will have a distinct channel for data communication. Therefore, the end-to-end delay is further minimized and the network throughput is increased.

The rest of the paper is organized as follows. Section 2 focuses on the recently proposed quorum-based MAC protocols. Section 3 provides a detailed description of QMMAC. Section 4 provides the result, and finally, Section 5 concludes this paper.

## 2. Related Work

In this section, we mainly focus on the recent protocols that are related to the focus of our protocol. First, we will discuss the quorum-based wakeup scheduling protocols. Then, we will review the quorum-based multichannel protocols.

### 2.1. Quorum-Based Wakeup Scheduling MAC Protocols

A quorum-based MAC protocol (QMAC) [12] is designed for corona-based WSNs to reduce node idle listening time according to the node load. This protocol uses the grid quorum system in which the grid size of each node is determined by its corona traffic load. In addition to using a different grid size to reduce the end-to-end latency, QMAC allows nodes to rely on several forwarders to forward their traffic to the sink, and thus the end-to-end latency is reduced even further. However, QMAC suffers from collisions when two nodes located at the same corona select the same forwarders and start simultaneously sending data at an intersecting quorum slot.

Another quorum-based MAC protocol (QTSAC) [13] is proposed to minimize the network delay by increasing the number of quorum slots based on the nodes’ remaining energy. The quorum slots in QTSAC are condensed at the node’s data transmission time. Each node then places the condensed grid into the original grid. QTSAC improves the network throughput while reducing the latency and extending the network lifetime. On the other hand, especially in the low traffic load area, increasing the number of quorum slots leads to a larger duty cycle which results in higher energy consumption. Furthermore, QTSAC protocol did not present a solution for collisions.

QueenMAC [14] is an energy-efficient MAC protocol that aims at decreasing nodes’ wake-up times. QueenMAC operates on multichannel for simultaneous data transmissions. In QueenMAC, nodes schedule their wakeup times using the dygrid quorum system. The dygrid quorum system is comprised of two groups of quorums: H-quorum and V-quorum. The dygrid quorum does not guarantee the intersections among the nodes belonging to the same group as they are not supposed to communicate. However, it guarantees that two neighbor nodes from different groups meet each other $k_{1}\times k_{2}$ times during *n* slots, where $k_{1},k_{2}$ are two integers derived from nodes’ traffic load. To reduce collisions, a channel assignment procedure that provides two-hop channel separation is conceived. However, a collision may happen when two nodes from the same corona select the same forwarders and start simultaneously sending data at an intersecting quorum slot. Nevertheless, the dygrid quorum system increases the number of intersections between neighbors, which decreases the forwarding delay.

TLS protocol [16] is an energy-efficient MAC protocol that utilizes the grid quorum system to schedule nodes’ wake-up times. Each node will decide its grid size based on its maximum traffic load. According to TLS, each node can calculate its traffic load based on the density of its communication range and its hop count. Since the node traffic load may change, each node can recalculate its traffic load, and whenever the node’s traffic load changes, the node can recalculate its active ratio and its suitable grid size. In order to find the next-hop forwarder, TLS proposed a path selection algorithm. The node sends a request to its next-hop neighbors to collect their remaining energy information. After the reply of the next-hop neighbors, the node will choose the next-hop neighbor with the highest remaining energy as its forwarder. By using the grid quorum system, this protocol guarantees at least two intersections between the node and its forwarders. However, a collision may happen when two nodes from the same corona choose exactly the same next-hop forwarder and start transmitting data simultaneously in the same slot.

### 2.2. Quorum-Based Multichannel MAC Protocols

Multichannel MAC protocols highly reduce the end-to-end delay by enabling multiple simultaneous successful transmissions between neighbor nodes. Mainly, there are two protocols that use the quorum system to share channels between nodes. 

MM-MAC [17] is a cyclic quorum-based multiple rendezvous multichannel MAC protocol. In this protocol, the time is divided into frames, and each frame is partitioned into two periods: control period and data period. The control period is divided into *n* control slots. A control slot can be either: a default slot or a switching slot. At the default slot, a node remains on its default channel for communication requests. At switching slots, a node can switch to its intended receiver’s default channel to start data transmission. In order for MM-MAC to enable communications between sensor nodes, the node’s switching slots must intersect with its receiver’s default slots. Each node selects a quorum set from the cyclic quorum system based on its ID and current frame ID. In this protocol, the quorum elements represent the node’s default slots. MM-MAC guarantees the intersection between the receiver’s default slots and the sender’s switching slots. The separation of the control and data periods reduces the collision probability between data packets and control packets. On the other hand, as a result of repeated notification packets exchange at the control period to indicate that the channel has been reserved, the energy consumption is increased. In addition, the fixed size of the data period imposes two nodes to go to their default channels and start the communication process again even if they still have more data packets.

MC-UWMAC [18] is a multichannel MAC protocol designed for underwater acoustic sensor networks. This protocol aims at avoiding collision without extra control packets exchange. According to MC-UWMAC, there is one common control channel, and $\frac{n\left(n-1\right)}{2}$ multiple data channels, where *n* is the maximum number of one-hop neighbors. The control channel time is partitioned into frames. Each frame is further partitioned into *n* slots. Each node is assigned a unique control frame slot in order to avoid collision in the control channel. A node, during its unique slot, exchanges RTS and CTS packets with its intended receiver. If the RTS and CTS exchange is successfully completed, the sender and its intended receiver switch to a unique reserved channel to communicate through. To do so, every node is allocated a set of data channels where each channel is reserved for data communication with an intended neighbor. To define nodes’ data channel sets, the singleton intersection quorum system is proposed where every two neighbors have a distinct common data channel. To assign a unique control channel slot and a unique quorum set, MC-UWMAC uses the grid-based partition to derive this unique number from the geographical coordinate of the node. For collision-free communication, all nodes in the network listen to the common control channel and update a meeting table that contains all in-progress communication. Moreover, this protocol uses more bit to reduce the delay. MC-UWMAC significantly reduces the collision probability without additional packet exchange required. Furthermore, it overcomes the hidden node and missing receiver problems thanks to the quorum-based slot allocation and meeting table. On the other hand, when the neighborhood size increases, the throughput and the energy efficiency decrease, and the meeting table become unmanageable. Therefore, this protocol is more suitable for sparse networks with a small neighborhood size.

## 3. QMMAC Protocol

### 3.1. Quorum Concept

A quorum concept is a useful concept that has been widely used in distributed systems for mutual exclusion [19] and replica control problems [20,21], in mobile ad hoc networks for location management [22], in WSNs for information dissemination [23] and data aggregation [24], and in underwater sensor networks for multichannel MAC protocol design [17,18]. A quorum system is defined as follows.

**Definition** **1.** *Given a universal set U={0,…,n−1}*, *a quorum system*Q*under Uis a collection of non-empty subsets of U, each called a quorum, which satisfies the intersection property: $\forall G,H\in Q;G\cap^{\;}H\neq \emptyset$.*

For example, Q={{1,3},{1,2},{2,3}} is a quorum system with three non-empty subsets under U={1,2,3}. There are several kinds of quorum systems, such as grid [25], cyclic [26], torus [23] and others [27,28,29].

### 3.2. Overview

**Figure 3:**
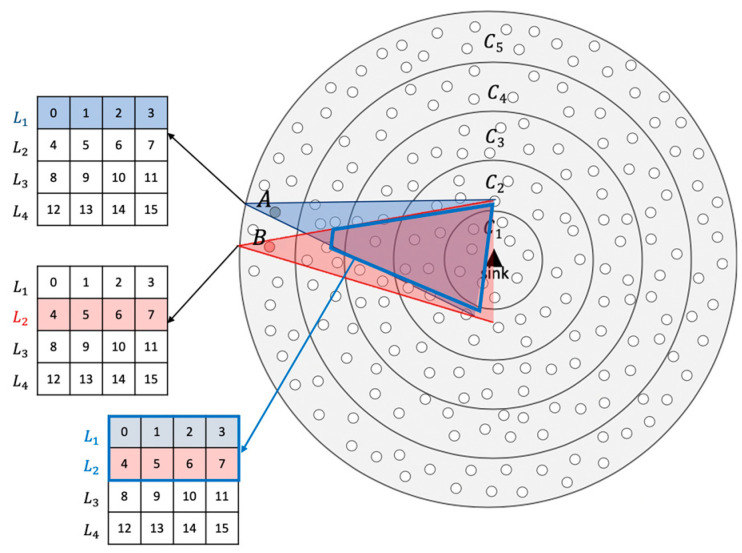
A five coronas network with an example of slots assignment for non-forwarder neighbors.

QMMAC is a corona-based energy-efficient multichannel MAC protocol, where nodes are arranged in coronas of equal width according to their hop counts from the sink and the sink is placed at the center of a circular field. A network of five coronas is illustrated in Figure 3. As shown in the figure, nodes in corona $C_{i}$ are *i* hops away from the sink. The idea of QMMAC is to allow each node and all its forwarders to wake up at the same time while avoiding collision and overhearing by keeping them separated using the multichannel feature. Specifically, our protocol consists of two procedures: (1) quorum-based wakeup scheduling and (2) quorum-based channel assignment.

First, QMMAC quorum-based wakeup scheduling is designed to assign each node a line from a n×s grid as its wakeup slots, and hence the idle listening will be reduced. Section 3.3.1 will describe how to calculate the appropriate values of n and s. Moreover, to reduce the end-to-end delay, any node will have exactly the same wakeup slots (line) with its next-hop forwarders. Furthermore, according to QMMAC, there will be zero intersecting wakeup slots between neighbor nodes that are not acting as next-hop forwarders for each other and hence collisions will be avoided. Thanks to our quorum-based wakeup scheduling, the end-to-end delay will be reduced as each node will wake up at exactly the same time as its potential forwarders. Indeed, despite the efficiency of the quorum-based wakeup scheduling in terms of collision and energy consumption, they introduce extra end-to-end delay as nodes need to wait for the appropriate wakeup slot to meet an intended receiver. That being said, although QMMAC mitigates such extra waiting delay by allowing a node to wake up at exactly the same time as its potential forwarders, overhearing and collision may occur between the node and its next-hop forwarders. To overcome such limitations and as a second main contribution of our protocol, we design a multichannel MAC protocol to separate forwarders communications without increasing the end-to-end delay. Section 3.4 will provide a detailed description of multichannel communication.

**Figure 4:**
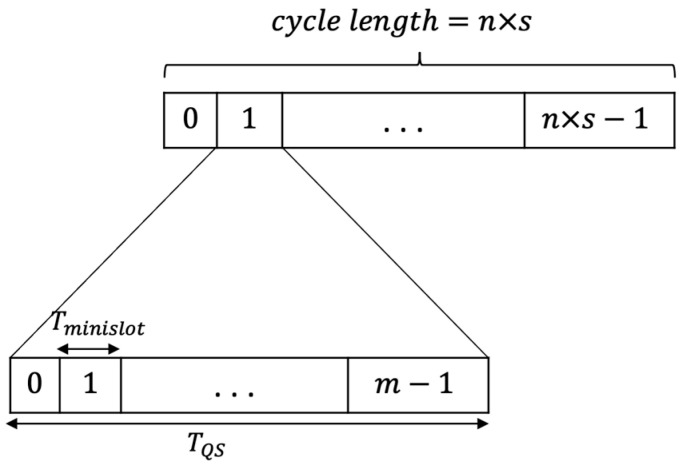
The quorum slot structure.

Our protocol’s main idea can be described as follows. QMMAC is built based on IEEE 802.15.4 with sixteen non-overlapping channels. According to our protocol, there are one shared control channel for handshaking and fifteen channels for data communications. The time at the control channel is slotted into n×s slots. Based on QMMAC wakeup scheduling, there are two types of slots: quorum and non-quorum. QMMAC will properly determine the sleeping and wakeup slots for every node, as described in Section 3.3. Every quorum slot is further split into m minislots. One minislot will be allocated to each node. A node with a data packet will attempt at its assigned minislot to transmit RTS to its next-hop forwarders. All next-hop forwarders should back off before replying with CTS. If the node and its forwarder successfully exchange RTS/CTS, they will switch to a unique channel for data communication. Figure 4 shows the quorum slot structure. QMMAC uses the singleton intersection quorum system proposed in [18] to generate different channel sets for the data channel assignment. Consequently, every quorum is a subset of channels that would be assigned to the node. Moreover, the intersection between two quorum sets is a different singleton. Thus, each node will have a unique data channel with each of its next-hop forwarders to communicate through. Based on the built channel sets, we design our channel assignment such that each node in a given neighborhood will be assigned a unique channel set and hence the collision between conflicting wakeup nodes are avoided. This channel assignment is described in more detail later in Section 3.4.1 and Section 3.5.1. In fact, QMMAC will achieve multiple successful simultaneous communications between conflicting forwarders thanks to the multichannel feature.

### 3.3. Quorum-Based Wakeup Scheduling

The aim of QMMAC wakeup scheduling is to reduce the energy consumption and the end-to-end delay. We propose the line quorum system in which each quorum set includes a line from a n×s grid. To begin, a node in the last corona will be randomly assigned a line as its wakeup time slots. The line quorum system assigns nodes within two-hop neighborhood different quorums to ensure zero intersecting slots between them. After that, the node’s next-hop forwarders will be assigned the same quorum (line) to minimize the end-to-end delay.

An example is shown in Figure 3. Node A and its upstream forwarders are assigned line *L_1_*, nodes in the blue sector in Figure 3. Node B, which is a neighbor of A, will wake up at *L_2_*, as illustrated in Figure 3. It should be noted that even if there is an intersection between A’s sector and B’s sector, as shown in Figure 3, the intersecting nodes will have a higher active ratio as they first wake up at slots 0, 1, 2, and 3 with node A and then wake up at slots 4, 5, 6 and 7 with node B. The next sections will explain how to define the appropriate grid size and how to allocate a unique line from the grid to nodes in the last corona nodes.

#### 3.3.1. Grid Size

To determine the grid size, we first calculate the number of lines n such that nodes in the last corona within two hops of each other are allocated unique lines to avoid collisions, and thus energy waste is minimized. Then we define the number of slots in each line s such that each node has enough wakeup time for its traffic.

**Figure 5:**
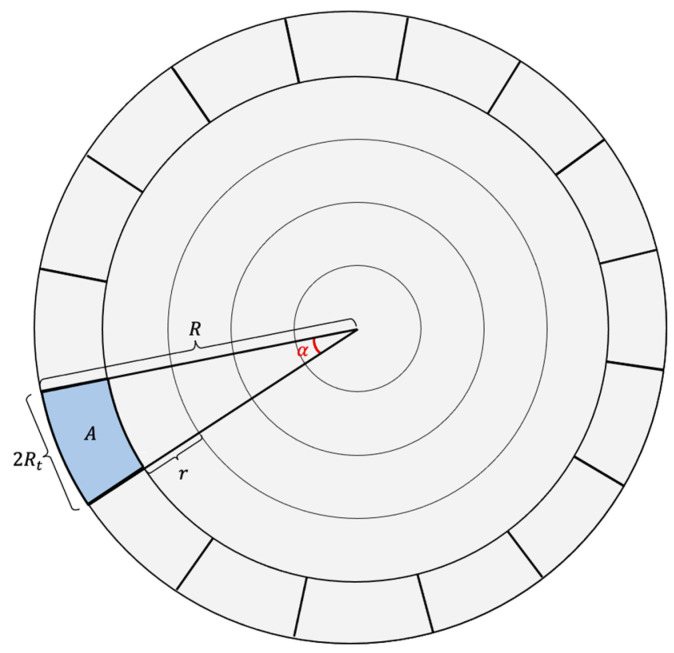
Partition of the last corona into regions of area A.

To estimate the number of lines, we divide the last corona into regions of area A representing a two-hop neighborhood, as shown in Figure 5, such that each node in area A will be assigned a distinct line. Accordingly, *n* is defined to be the average number of nodes in a given two-hop neighborhood. The area A of the different regions should be carefully determined to avoid two neighbor nodes in different regions will end up with the same line assignment and hence collisions will happen. To do so, we suggest to determine A such that the outermost boundary of the last corona is partitioned into arcs of size $2R_{t}$ as shown in Figure 5. By doing so, each region will be representing a neighborhood where all nodes are listening to each other and hence should be assigned each a different line between 0 and n−1 in order to avoid collisions during their conflicting transmissions. Accordingly, n  can be simply derived as

(1) n=A×d

where d is the network density that can be derived as follows:

(2) $$d=\frac{N}{\pi R^{2}}$$

where N is the number of nodes in the network and R is the field radius. Now, in order to find A, let us first derive the angle α of the sector with the outermost arc equals $2R_{t}$.

(3) $$\alpha =\frac{2Rt}{i_{max}*r}$$

where $i_{max\;}$ is the number of coronas and r is the corona radius. Thus, A can be simply written as

(4) $$A=\frac{\alpha }{2}r^{2}\left(2i_{max}-1\right)=R_{t}r\left(2-\frac{1}{i_{max}}\right)$$

**Figure 6:**
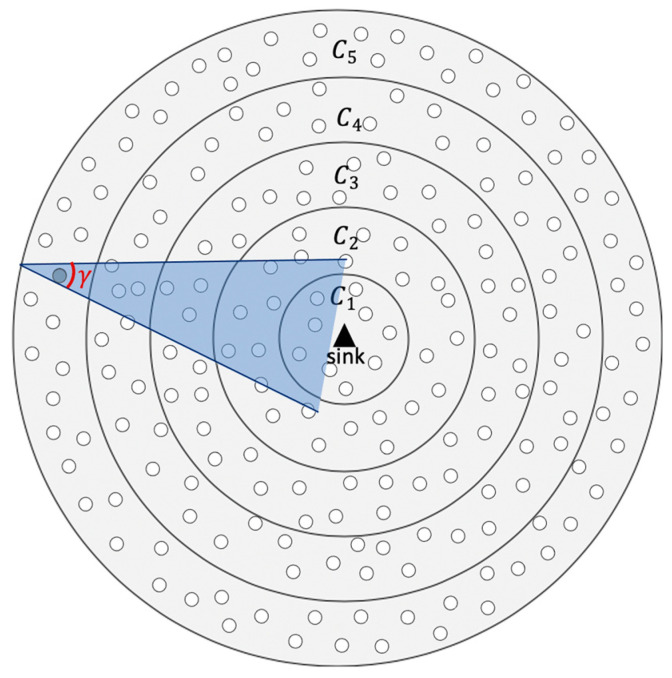
Gamma sector.

Next, we need to define the number of slots s in each line properly such that each line has enough slots for wakeup nodes on each sector to transmit their data. Consequently, s can be expressed as follows

(5) $$s=N_{sector_{avg}}\times H_{avg}$$

where $N_{sector_{avg}}$ is the average number of nodes in the gamma sector which represents the average number of nodes that wake up at the same line. The gamma sector is shown in Figure 6. $N_{sector_{avg}}$ can be defined as follows:

(6) $$N_{sector_{avg}}=\frac{\gamma }{2}\;R^{2}d\;$$

$H_{avg}$ is the average number of hops in the gamma sector that can be derived as follows

(7) $$H_{avg}=\frac{\sum_{i=0}^{i_{max}}N_{sector_{c}{}_{i}}\times i}{N_{sector_{avg}}}$$

where $N_{sector_{c}{}_{i}}$ is the number of nodes in the intersection of corona i and the gamma sector and can be derived as follows

(8) $$N_{sector_{c_{i}}}=\frac{\gamma }{2}\;r^{2}\left(2i_{max}-2i+1\right)$$

Finally, let us derive the angle γ,

(9) $$\gamma =\frac{A_{unit}}{r^{2}}\times 2$$

where $A_{unit}$ is the area unit that contains one node and can be derived as follows

(10) $$A_{unit}=\frac{1}{d}$$

#### 3.3.2. Line Assignment

**Figure 7:**
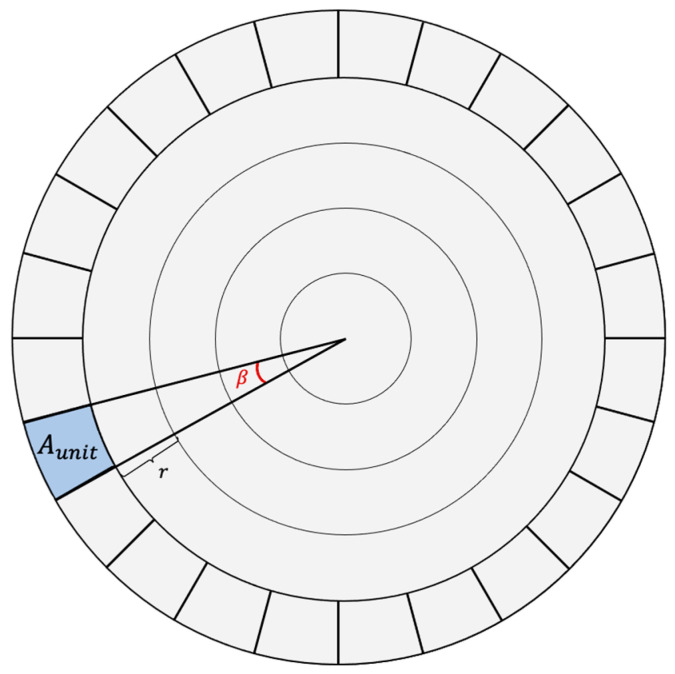
Partition of the last corona into sectors with angle β.

After the grid size is determined, now, the issue is how to assign a distinct line l from the grid to the last corona nodes such that two-hop conflict free line assignment is guaranteed. To accomplish this, the circular area will be divided into equal sectors, with one node on each. As illustrated in Figure 7, we divide the area to equal-size sectors of angle β. Referring to Figure 7, let $\left(x_{u\;},y_{u\;}\right)$ is node u geographical coordinates and $\left(x_{s\;},y_{s\;}\right)$ is the sink geographical coordinates then

(11) $$A_{unit}=\frac{1}{d}=\frac{\beta }{2}r^{2}\left(\;2i_{max}-1\right)$$

Next, let q represents the sector’s number in which node u is placed. Accordingly, q is a unique number between 0 and $\frac{2\pi }{\beta }$, the maximum number of sectors, and can be derived as

(12) $$q=quotient\left(\frac{\varphi }{\beta }\right)$$

where the angle φ is between the positive *x*-axis and node u and can be defined as

(13) $$\varphi =\{\begin{array}{ll} \tan^{-1}\left(\frac{\left|\;y_{u}-y_{s\;}\right|}{\left|\;x_{u}-x_{s\;}\right|}\right) & if\;\begin{array}{l} x_{u}-x_{s\;}>0\\ \;y_{u}-y_{s\;}>0 \end{array}\\ \pi -\tan^{-1}\left(\frac{\left|\;y_{u}-y_{s\;}\right|}{\left|\;x_{u}-x_{s\;}\right|}\right) & if\;\begin{array}{l} x_{u}-x_{s\;}<0\\ \;y_{u}-y_{s\;}>0 \end{array}\\ \pi +\tan^{-1}\left(\frac{\left|\;y_{u}-y_{s\;}\right|}{\left|\;x_{u}-x_{s\;}\right|}\right) & if\;\begin{array}{l} x_{u}-x_{s\;}<0\\ \;y_{u}-y_{s\;}<0 \end{array}\\ 2\pi +\tan^{-1}\left(\frac{\left|\;y_{u}-y_{s\;}\right|}{\left|\;x_{u}-x_{s\;}\right|}\right) & if\;\begin{array}{l} x_{u}-x_{s\;}>0\\ \;y_{u}-y_{s\;}<0 \end{array} \end{array}$$

According to the above equations, Node u can be represented by the unique number q. Consequently, a unique line between 0 and n−1 can be assigned to each node in a given area A as follows

(14) l=q mod n 

### 3.4. Quorum-Based Multichannel Communications

QMMAC is built based on IEEE 802.15.4. There is one shared control channel for handshaking and fifteen channels for data communications. The time at the control channel is divided into n×s slots. Every quorum slot is further split to m minislots. One minislot will be assigned to each node for RTS/CTS packets exchange. A node exchanges RTS and CTS packets with its next-hop forwarders. If the node and one of its forwarder exchange RTS/CTS successfully, they will switch to a unique dedicated data channel. In the following sections, we describe the channels assignment and control channel minislots assignment.

#### 3.4.1. Channel Set Assignment

QMMAC utilizes fifteen channels for data communications. Each node will have a distinct channel with each one of its next-hop forwarders to communicate through. To do so, first, we will use the singleton intersection quorum system proposed in [18] to build the different quorum sets. Then, unlike the work in [18], an assignment process will be done at the initialization phase such that each node in a given neighborhood will be assigned a unique and different channel set.

In the singleton intersection quorum system, every quorum is a subset of channels that would be allocated to the node. Moreover, the intersection between two quorum sets is a different singleton. Thus, every node with each of its forwarders will have a distinct and unique data channel for data communication. The singleton intersection quorum system is defined as follows:

**Theorem** **1.** *Ref. [18], Given G, the system $Q=\{S_{1},\ldots ,\;S_{G}\},$* *where*

$$\{\begin{array}{c} \forall \;1\;<\;j\;\leq \;G\;;\;\mathrm{card}\left(S_{j}\right)=G-1\;,\;S_{1}=\left\{1,2,\ldots ,G-1\right\}\;,\\ \mathrm{and}\;S_{j}=\{\;{\left(S_{1}\right)}_{j-1},\;\ldots \;,{\left(S_{j-1}\right)}_{j-1},{\left(S_{j-1}\right)}_{G-1}+1,\\ \;\ldots \;,{\left(S_{j-1}\right)}_{G-1}+(\;M\;-\;j\;)\;\}\\ {\left(S_{w}\right)}_{p}\;refers\;to\;the\;p^{th}\;element\;of\;S_{w} \end{array}$$

*is a quorum system under* U={1, . . . , G} *where G is the number of distinct elements and equals to* $\frac{k\left(k-1\right)}{2}$ *where k is the number of nodes that will be sharing the quorum system.*

We built our quorum system as a unique singleton intersection with 15 data channels (G=15) and six neighborhood sizes. Hence, each node should have a maximum of five next-hop forwarders. Thus, QMMAC has six channel sets, and each one consists of five elements as follows:

$$\begin{array}{l} S_{1}=\left\{1,\;2,\;3,\;4,\;5\right\}\\ S_{2}=\left\{1,\;6,\;7,\;8,\;9\right\}\\ S_{3}=\left\{2,\;6,\;10,\;11,\;12\right\}\\ S_{4}=\left\{3,\;7,\;10,\;13,\;14\right\}\\ S_{5}=\left\{4,\;8,\;11,\;13,\;15\right\}\\ S_{6}=\left\{5,\;9,\;12,\;14,\;15\right\} \end{array}$$

Consequently, each node will be allocated one of the above channel sets. To assign the channel sets, the last corona’s nodes will choose one channel set randomly. Next, every node will be in charge of assigning a channel set to each one of its selected forwarders. After a forwarder is assigned a channel set, it will by its turn allocate channel sets to its next-hop forwarders, and so on. To guarantee the unique assignment, the node will assign the channel sets that are not selected by its neighbors to its next-hop forwarders. This process is described in more detail later in Section 3.5.1.

#### 3.4.2. Control Channel Minislot Assignment

Every node will be assigned one minislot for RTS/CTS packets exchange. We need to define the minislots number m to assign a different minislot to conflicting neighbor nodes. In the gamma sector, shown in Figure 6, nodes in two successive coronas represent a neighborhood where nodes are conflicting. Accordingly, to allow each node in the gamma sector to have a distinct unique minislot, we can overestimate m to be the number of nodes in *C_1_* and *C_2_*, which contain most of the nodes in the gamma sector. Thus, collisions are further reduced. m can be defined as

(15) $$m=N_{sector_{c_{1}}}+N_{sector_{c_{2}}}$$

Finally, node u can choose its minislot as follows

(16) $$MS_{u}=node_{ID}\;mod\;m$$

### 3.5. Protocol Description

#### 3.5.1. Initial Configuration

QMMAC is designed for corona-based WSNs where nodes are organized into equal-width coronas around the sink. In these networks, nodes in $C_{i}$ rely on nodes in $C_{i-1}$ to transmit their data towards the sink. QMMAC allows nodes to rely on more than one next-hop forwarders such that they wake up at the same time. Thus, the probability of a node to meet a forwarder is increased and the delay is reduced. At the initialization phase, the next-hop forwarders selection will take place. This selection process will be done using MEMBER_SOLICITATION, MEMBER_SOLICITATION_REPLY, and MEMBER_NOTIFIY control packets. First, the node will broadcast a MEMBER_SOLICITATION packet. Next-hop nodes will reply with MEMBER_SOLICITATION_REPLY that contains their information (such as node coordinates). After receiving the next-hop replies, the node will choose its forwarders and inform them using a multicast MEMBER_NOTIFIY packet.

According to the channel set assignment, wakeup nodes in a neighborhood should be assigned one of the six channel sets to avoid collision on the fifteen data channels. That is why the next-hop forwarders selection needs to be restricted to a limited number of forwarders such that in a given neighborhood no more than six neighbor nodes will be waking up at the same time. Consequently, the number of forwarders can be defined as follows

(17) F=5−(p+1)−w

where  w is the number of downstream nodes served by the node and p is the number of neighbor nodes that wake up at the same time. To increase this number and allow more reuse of the channel sets, the node will choose the most distant nodes from each other (using the geographical coordinates provided in the reply packet) as its next hop forwarders, so the probability that they are neighbors is reduced and hence the p value is reduced and thus the number of forwarders F is increased.

**Figure 8:**
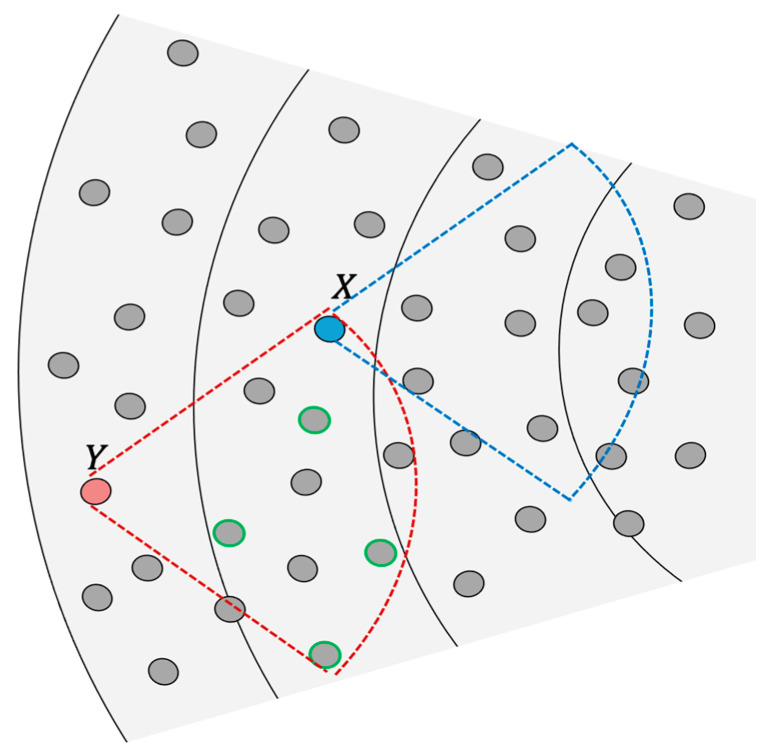
Forwarders selection.

For example, as shown in Figure 8, initially, node Y will select a random channel set. Then, using the aforementioned forwarders selection process, node Y that is in the outmost corona $C_{5}$ will send MEMBER_SOLICITATION packet to next-hop neighbors. Next-hop nodes will reply with MEMBER_SOLICITATION_REPLY. After that, node Y will select the farthest five nodes from each other from the nodes that replied as forwarders where w=0 and thanks to QMMAC line assignment p=0. Node Y will assign each one of them a channel set and send its choice in a multicast MEMBER_NOTIFIY packet such that the selected forwarders know which channel sets are assigned to their neighbors. After node Y sends its notification packet, node X that is in $C_{4}$ will start its selection process. First, node X knows which one of its neighbors serves node Y and which channel sets are assigned to them. Assume that node X is serving node Y only and have one neighbor serving node Y, then node X will send a solicitation packet, wait for replies and then choose the farthest two next-hop nodes (p=1 and w=1) and assign them their channel sets. Finally, it will send the notification packet, and the next-hop selected forwarders will similarly start their selection.

Assume that the sink listens to the sixteenth channel simultaneously. Another channel assignment will be done at the initialization phase to assign nodes in $C_{1}$ a channel to use when communicating with the sink. First, at the forwarders selection process, when nodes in $C_{2}$ assigns sets to their forwarders nodes in $C_{1}$, they send their used channel with downstream nodes. Nodes in $C_{2}$ will have at most five channels used with downstream nodes. So their forwarders have at least 11 channels to use with the sink. Nodes in $C_{1}$ will report these channels to the sink and the sink will check the available channels and assign one of these channels to these nodes. It should be noted that the sink will manage requests one by one and each time it will eliminate the assigned ones.

**Figure 9:**
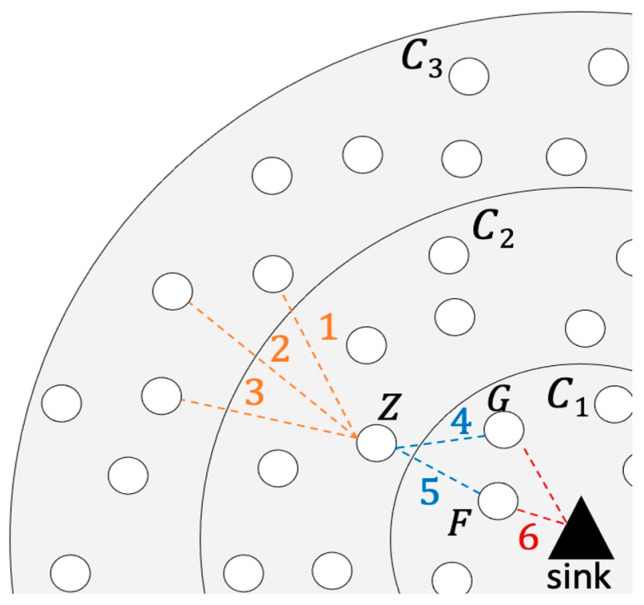
Channel assignment for nodes in $C_{1}$.

For example, as shown in Figure 9, if node Z wakeup at line 1, and use channel 1,2, and 3 for downstream communications, their upstream forwarder (node F and node G) will have 11 non-used channels (5–15 for node F and 4, 6–15 for node G) available to use with sink. If node F asks for channel and the sink assigns channel 6 to node F, then the sink will have 14 channels left (1–5, 7–15) to assign for nodes waking up in line 1. Then, node G can be assigned one of the 10 non-used channels (4, 7–15), where channel 6 is assigned to node F.

It is true that nodes need the next-hop nodes’ locations to properly select its next-hop forwarders. Moreover, the sink needs to know the used channels by nodes in C_2 to assign nodes in C_1 one of the available channels to use when communicating with the sink. That being said, we want to point out that this information namely nodes’ locations and used channels by $C_{2}$ nodes, will be exchanged once at the initialization phase with the next-hop forwarders selection packets. More precisely, the locations will be included in MEMBER_SOLICITATION_REPLY packets and the used channels will be included in MEMBER_NOTIFIY packets of $C_{2}$ nodes. It is undeniable that the initialization phase requires time and consumes energy, but the exchanged information will save much more energy and time later. QMMAC is designed to reduce as much as possible of the energy wastage, decrease the end-to-end delay as well as improve the network throughput which make it well suited for WSNs. Indeed, the next-hop forwarders selection process will reduce the end-to-end delay where the probability of a node to meet a forwarder is increased. Moreover, the choice of the most distant nodes from each other (using the next-hop nodes’ locations) will allow more reuse of the available channels and mitigate the collisions among forwarders; thus, the energy wastage and the delay will be decreased. Furthermore, the use of the available channels to communicate with the sink will highly reduce the collision in $C_{1}$ and hence more energy will be saved. Additionally, thanks to the minislots assignment and the energy-based backoff, which avoids or even mitigates the control packets collisions, QMMAC further decreases the end-to-end delay and the energy consumption.

#### 3.5.2. Sender and Receiver Behaviors

All nodes in the network will wake up according to QMMAC quorum-based scheduling and listen to a common control channel. The time is slotted to n×s slots and every slot is further divided into m minislots. The minislots are assigned to nodes as described earlier in Section 3.4.2. In order to switch to the appropriate data channel, the sender sends an RTS to its next-hop forwarders. The next-hop forwarders will back off and send CTS. Consequently, the minislot time can be defined as

(18) $$T_{minislot}=T_{RTS}+T_{CTS}+T_{backoff}+2\times T_{prop}$$

where $T_{RTS}$ and $T_{CTS}$ are the transmission times of RTS and CTS packets, $T_{prop}$ is the maximum propagation time and $T_{backoff}$ is the maximum backoff time. The RTS and CTS packets contain the estimated end time of communication $T_{estim\_end}$ to inform their neighbors and thus they avoid collision. $T_{estim\_end}$ is based on the number of packets to be sent and is calculated as follows

(19) $$T_{estim\_end}=T_{start\_next\_slot}+numPackets\times T_{data}+T_{ack}+2\times T_{prop}\;$$

where $T_{start\_next\_slot}\;$ is the start time of the next quorum slot and $T_{data}$ and $T_{ack}$ are the transmission times of data and acknowledgement packets. A node will attempt to send all packets in its queue. However, nodes will be limiting their transmission in the wakeup line/s in order to conserve energy and avoid collisions. Thus, the number of packets, numPackets, can be calculated as

(20) $$numPackets=\{\begin{array}{c} \begin{array}{c} \begin{array}{c} \begin{array}{c} \begin{array}{cc} \frac{QS_{remaining}\times T_{slot}-T_{ack}}{T_{data}}, & \;\;\;\;\;\;\;\;\;\begin{array}{c} queueSize\times T_{data}+T_{ack}\\ >QS_{remaining}\times T_{slot} \end{array}\\ queueSize, & Otherwise \end{array} \end{array} \end{array} \end{array} \end{array}$$

where $QS_{remaining}$ is the number of remaining wakeup slots in the line.

***Sender behavior:*** At wakeup slots, a node having a packet to send will calculate the number of packets to send and $T_{estim\_end}$. Then, it will send a RTS that includes $T_{estim\_end}$ on its minislot $MS_{u}$ to its next-hop forwarders. If the next-hop forwarders successfully received the RTS, then they will back off and send a CTS. Nodes maintain a ‘meeting table’ that contains the in-progress communications with the used data channels along with their estimated end times. When a sender node receives the CTS, the sender will check if the packet is destined to it. If so, it checks the meeting table. If the pre-selected (see Section 3.4.1) data channel is busy, the sender will cancel its transmission. If the data channel is free, the sender waits until the end of the current slot before switching to the dedicated data channel. During the slot time, when a node receives a packet, the node will keep the meeting table updated to avoid collisions at the data channel. The sender will proceed sending the number of packets announced during the handshaking at the data channel. Then after the estimated end time, the receiver sends an acknowledgment with the identifiers of the successfully received packets. When the ACK is received, the sender will read the ACK and try to resend the non-received packets at the next wakeup slot. If the ACK is not received, the sender assumes all the packets were not received and tries to resend them at the next wakeup slot.

***Receiver behavior:*** at wakeup slot, a node listens to the control channel, if it does not have packet to send. If a RTS is received, the receiver checks if the packet comes from its downstream node. If so, before sending the CTS, the receiver checks if the channel is not busy. Then, it should back off a random time *B*. The backoff time *B* is based on the node’s residual energy. First, a parameter P will be defined as

(21) $$P=\frac{Residual\_Energy}{Initial\_Energy}$$

**Figure 10:**
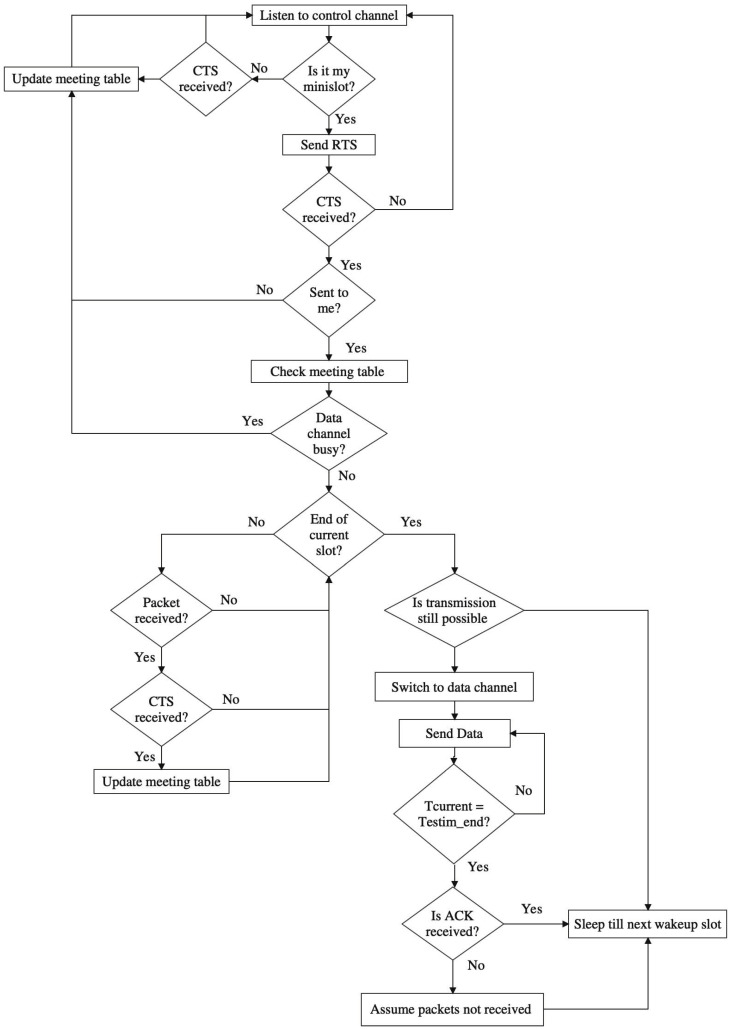
Sender behavior.

**Figure 11:**
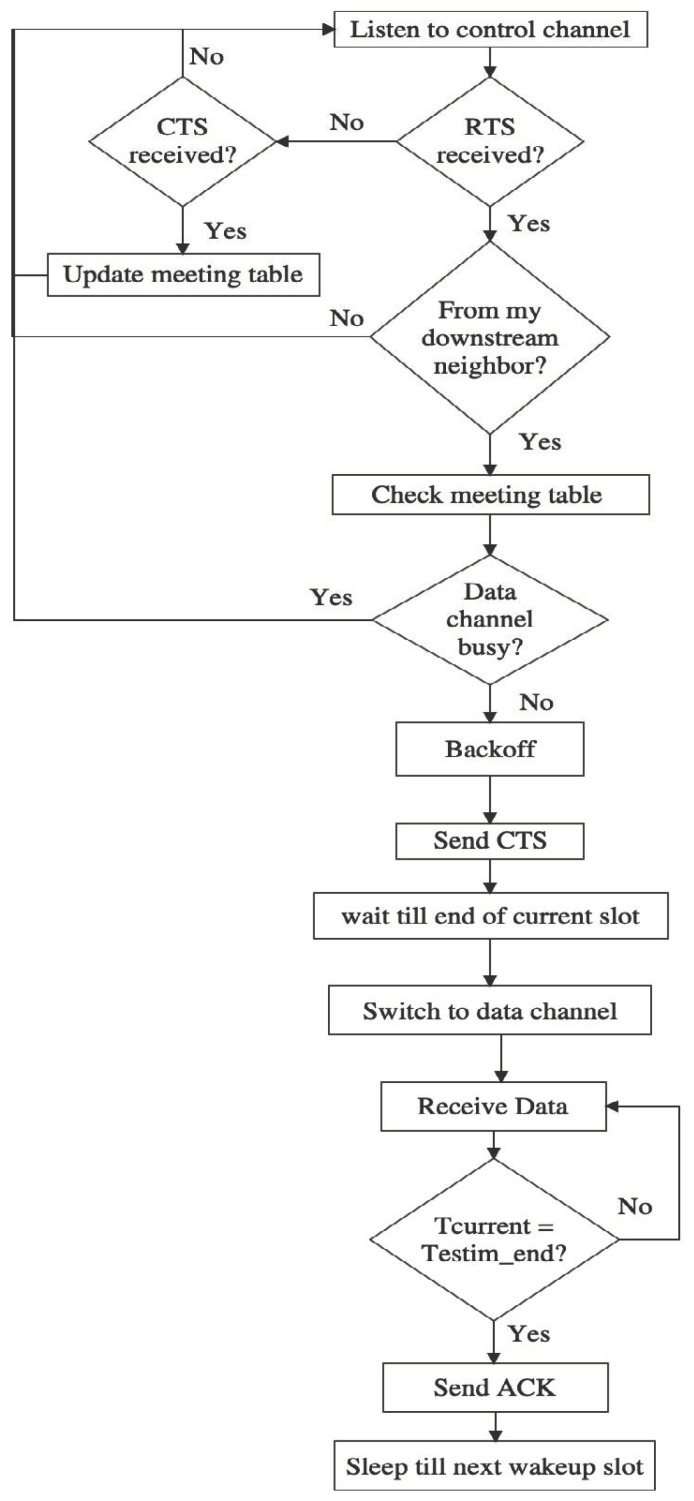
Receiver behavior.

If P≥0.5, which means that the node has more than half of its initial energy, the node set the backoff time at the first half of the contention window and hence the backoff interval is set equal to [0, … , Max_Value×(1−p) ]. Otherwise, the backoff interval is set at the second half, [ Max_Value×(1−p), … , Max_Value]. This way, the next-hop forwarder with higher remaining energy will be more eligible to reply first and hence the energy consumption is balanced among nodes. After a receiver node sends the CTS, it waits till the end of the current wakeup slot, then switches to the data channel. Note that during the wakeup slot if a potential next hop forwarder receives a CTS destined to its downstream node then it will refrain from sending its own CTS as the sender is well served by a forwarder with higher remaining energy. At the dedicated data channel, the receiver will listen to the channel without sending an acknowledgment till the expiration of $T_{estim\_end}$. After that, it will send the acknowledgment with the IDs of non-received packets in case of lost packets. Figure 10 and Figure 11 demonstrate the sender and receiver behaviors.

## 4. Performance Evaluation

**Figure 12:**
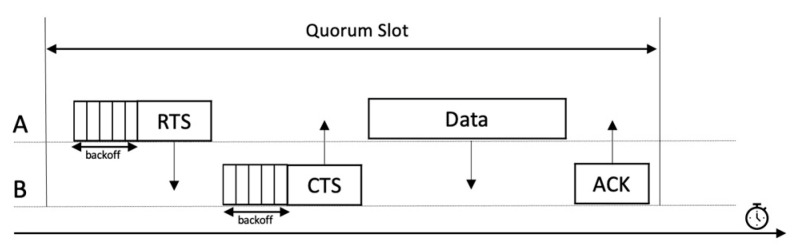
RTS/CTS/DATA/ACK dialog.

OMNET++ simulator [30] is used to implement and evaluate the performance of QMMAC. Two single-channel protocols are also implemented: the grid quorum system-based protocol (GridQS) where a 4×4 grid is used, and the single channel version of our QMMAC where QMMAC line quorum system is used. In GridQS, every node randomly chooses a raw and a column as its wakeup time slots. Figure 12 shows the four-way: RTS/CTS/DATA/ACK dialog used by the single-channel protocols for communication. It is true that the comparison with a single channel protocol may seems unfair, but it is worth comparing with in order to assess the performance of the multichannel feature. In our simulations, we want to evaluate the performance of the sleep wakeup scheduling as well we want to evaluate our contribution of using the multichannel feature to decrease the collisions between the wakeup conflicting nodes and to improve the network performance in terms of end-to-end delay and throughput. Moreover, the multichannel scheme is not guaranteed to perform perfectly in all scenarios as it has its own challenges such as the missing receiver, the hidden terminal problem, network partitions, etc. [31,32]. For these reasons, we compare our protocol QMMAC with the single-channel protocols. We implement and evaluate QMMAC, first, under regular topology that contains four-35 m coronas where 72 nodes are placed in 8 sectors. Second, under random topology where 60 nodes are randomly distributed in four coronas. The main simulation parameters are listed in Table 1. We use six metrics in our performance evaluation: energy per bit, throughput, end-to-end delay, data collision probability, data burst size and successful delivery ratio. The simulations show that the protocols have a quite similar behavior in the random topology as their behavior in the regular topology since the random topology results are averaged over many random topologies.

**Figure 13:**
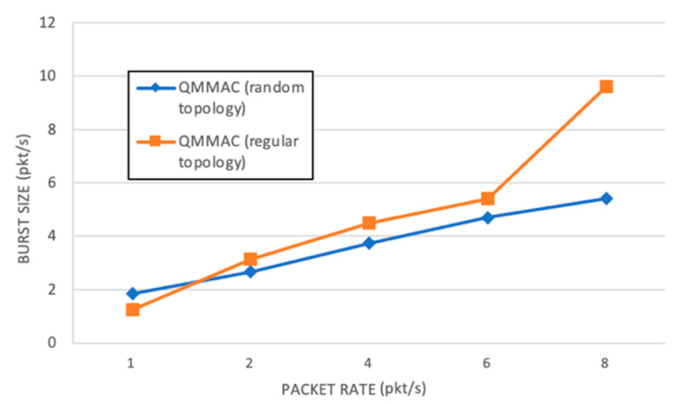
Burst size.

QMMAC allows nodes to send multiple data packets once it succeeds handshaking with one of its forwarders. Thus, the throughput is increased and the end-to-end delay is reduced. The burst size is defined as the average number of data packets transmitted per handshaking (RTS/CTS). Figure 13 shows the average burst size of QMMAC in both topologies. With the increase of the traffic rate, the burst size is increased as the nodes have more data packet in their queues.

**Figure 14:**
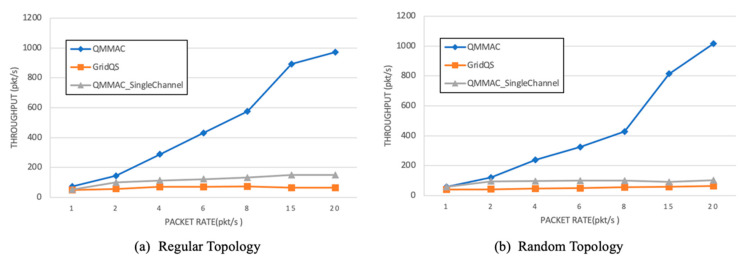
Throughput.

Figure 14 shows the average throughput as function of the traffic rate. The throughput is the amount of data packets successfully received by the sink during the simulation time. Thanks to the line quorum system, QMMAC shows better throughput in single and multichannel as it guarantees that a node meets its next-hop forwarders at its wakeup times to transmit its packets. QMMAC throughput is increased with the traffic rate, as shown in Figure 14. Indeed, QMMAC achieves much better throughput than other protocols by utilizing multiple channels for data communications. Furthermore, the throughput is further increased by utilizing the burst feature.

**Figure 15:**
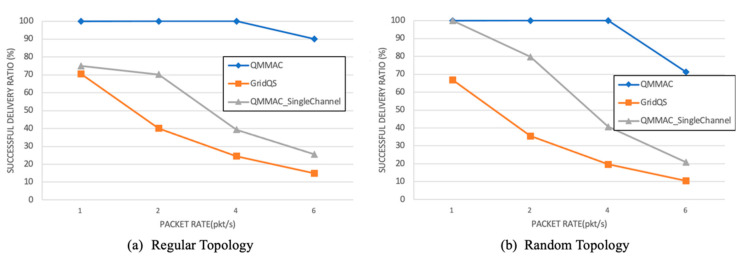
Successful delivery ratio.

Figure 15 depicts the successful delivery ratio as function of the traffic rate. The successful delivery ratio is the number of packets successfully received by the sink to the number of packets sent by all nodes. The delivery ratios of GridQS and QMMAC-single channel are low due to the increase of control packets collisions with the increase of the traffic rate, and thus the packets could not be delivered to the sink as a successful handshaking is needed for every data packet. For nodes running QMMAC, the average delivery ratio is 97% not only because of the use of the multichannel and burst features but also thanks to the minislots assignment and the energy-based backoff, which avoids or even mitigates the control packets collisions. 

**Figure 16:**
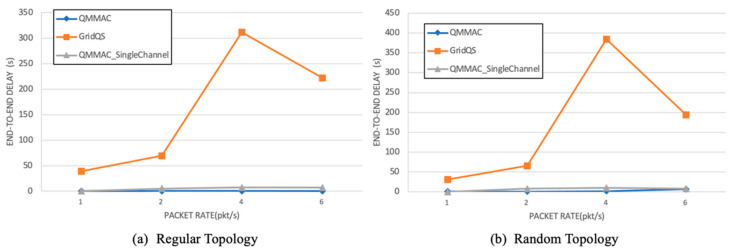
End-to-end delay.

**Figure 17:**
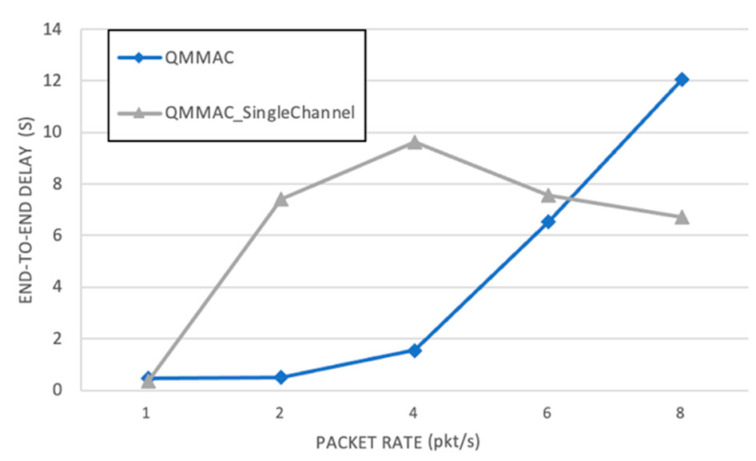
End-to-end delay for QMMAC and QMMAC-single channel.

Figure 16 illustrates the end-to-end delay as function of the traffic rate. As simultaneous data communications can be handled on different data channels, QMMAC achieves a much lower end-to-end delay. Besides, while the node transmits its packet to its forwarders, other forwarders can forward their packets to their forwarders which significantly reduces the end-to-end delay. Since all nodes are guaranteed to reach their forwarders during their wakeup time, the line quorum system not only reduces the end-to-end delay, but it also improves the throughput. In fact, as the full intersection is guaranteed between a node and its next-hop forwarders, the node does not have to wait for the intersecting quorum slot, and thus the end-to-end delay is greatly reduced. In the random topology, as shown in Figure 17, QMMAC single channel shows approximately the same end-to-end delay as the multichannel one or even better (at 8 packet/s). The reason behind this is that the delay is calculated for a small number of successfully received packets by the sink which most probably are generated by the first inner coronas nodes. Indeed, as shown in Figure 15, for a high traffic rate, the delivery ratio for single channel QMMAC is so low since the network is clogged with collisions. Consequently, only nodes in the first coronas will succeed to deliver their data packets to the sink. Thus, those received data packets have a reduced end-to-end delay as they are mostly emanating from nodes that are very close to the sink. As opposed, the multichannel QMMAC which achieves very high packet delivery ratio (as shown in Figure 15) where far nodes in the outermost coronas will succeed to deliver their packets to the sink. 

**Figure 18:**
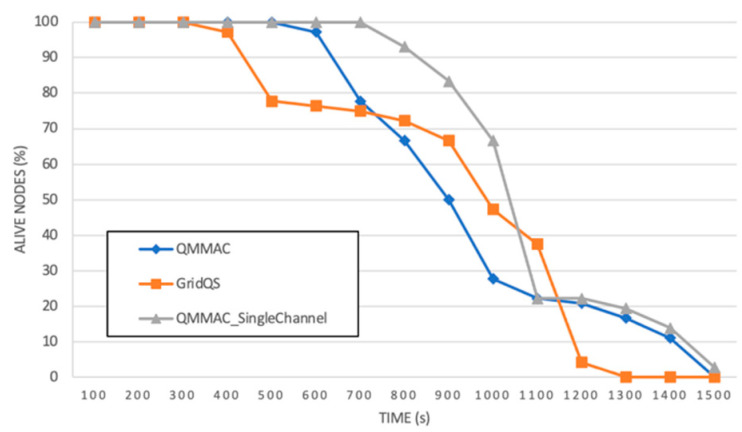
Alive Nodes.

To assess the performance of QMMAC in terms of network lifetime, we first observe the number of alive nodes in the network over 1500 s simulation time where sensor nodes have initial energy of 50 J. As shown in Figure 18, multichannel and single-channel QMMAC extended the network lifetime. At simulation time of 1300 s, all nodes of GridQS are running out of energy. Whereas, for both versions of QMMAC, all nodes run out of energy at simulation time 1500 s.

**Figure 19:**
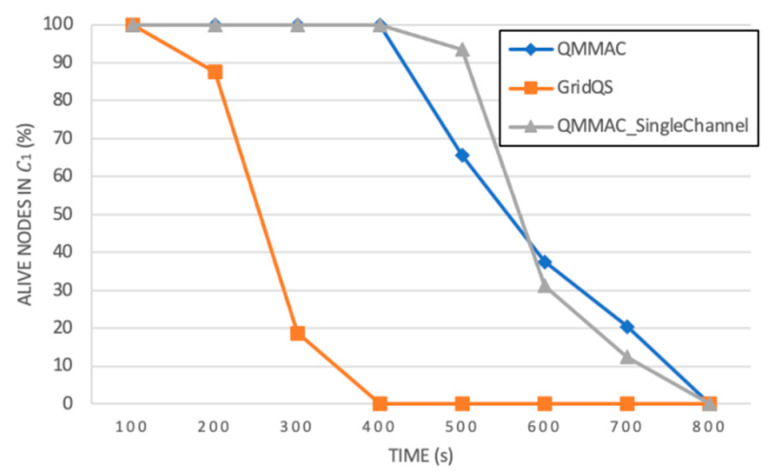
Alive nodes in $C_{1}$.

Let us now define the network lifetime as the time till nodes in $C_{1}$ die. Accordingly, we observed the alive nodes in $C_{1}$ for all protocols. As shown in Figure 19, all nodes in $C_{1}$ of GridQS die at time 400 s; hence the network will stop functioning. It should be noted that at time 400 s all nodes running QMMAC (multichannel and single-channel) are still alive. As shown in the figure, QMMAC extends the lifetime of $C_{1}$ nodes not only by reducing the idle listening, overhearing and collision, but also by balancing the energy between forwarder nodes using the energy-based backoff.

**Figure 20:**
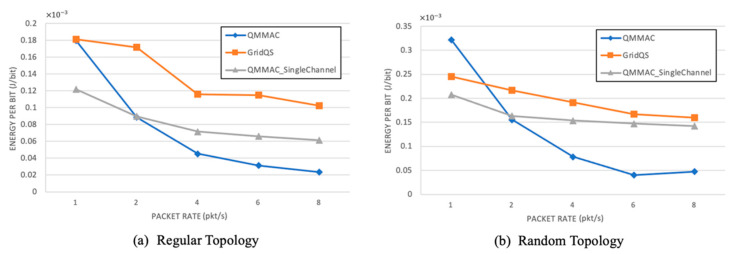
Energy per bit.

The average consumed energy per successful bit is shown in Figure 20. As expected, for all the protocols, the throughput is increasing and hence the energy efficiency is increasing with the traffic rate. Most importantly, QMMAC is more energy efficient than GridQS and QMMAC single channel. In fact, according to QMMAC, with increased traffic rate, the burst size is increasing too; therefore, more data packets are transmitted per single handshaking exchange. Consequently, more packets will be delivered with the same consumed energy for a single RTS/CTS exchange. At 1 packet/s, QMMAC is consuming more energy than the other protocols. Indeed, in GridQS and QMMAC single channel, a node needs to wake up at one fifth of the slot duration to exchange RTS and CTS, and then go back to sleep if no data communication is taking place. Unlike that, in QMMAC, the node will be awake during the whole wakeup line to update its meeting table to avoid collision, and thus more energy is consumed. That being said, this further energy consumption due to idle listening will be compensated when the traffic rate increases as it will be beneficial to avoid collisions, and hence we end up with higher energy efficiency.

**Figure 21:**
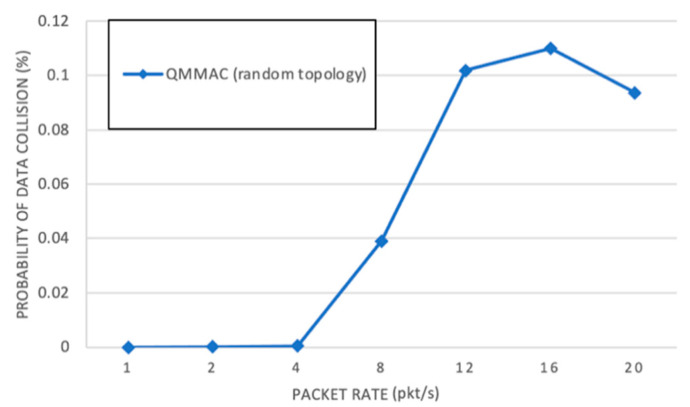
Data collision probability.

**Figure 22:**
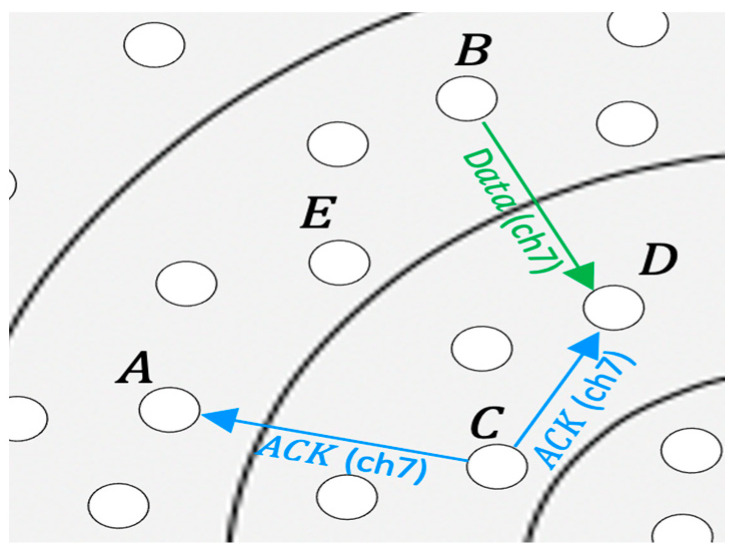
Data collision example.

Figure 21 shows the collision probability on data channels as a function of the traffic rate. By assigning wakeup neighbor nodes a unique slot and a unique channel, QMMAC tries to provide collision-free communications. However, when two non-neighbor downstream nodes, which use the same channel set, assign the same channel set to two neighbor nodes, a collision may occur at the data channel. These two nodes’ packets may collide if and only if they miss each other CTSs. If a node is busy transmitting on a data channel while its neighbors transmit their CTSs on the shared control channel, the node can miss their CTSs. The following scenario even very unlikely, may occur. Figure 22 shows an example of the unlikely data collision. Node A and B non-neighbor nodes are both assigned channel set 4. They allocate channel set 2 to their next-hop forwarder nodes C and D, respectively. Suppose D is a forwarder for node E and nodes C and D are neighbors. If node A and node C successfully handshake and agree to communicate for the next four slots, while E and D communicate on channel 9, node D will be unaware of their communication. After node D completes its transmission and back to the control channel, if nodes D and B successfully exchange RTS/CTS, they may go to channel 7 for data transmission before C and A complete their transmission. Accordingly, at node D, node C acknowledgment may collide with node A data packet. As shown in Figure 21, our protocol achieves extremely low collision probability since the above-described scenario is infrequent to occur.

## 5. Conclusions

In this article, we proposed a quorum based multichannel MAC protocol, QMMAC. QMMAC aims at decreasing the end-to-end delay and energy wastage and thus improving the throughput as well as the network lifetime. By utilizing the quorum concept, first, we propose a quorum-based wakeup scheduling that uses the line quorum system to minimize the end-to-end delay by allowing nodes to wake up at exactly the same time as its potential forwarders. Moreover, the useless overhearing, idle listening and collisions are avoided where nodes that are not acting as forwarders for each other wake up at a completely different time. Furthermore, to achieve the maximum energy efficiency and the minimum end-to-end delay, we defined the appropriate quorum size and proposed a quorum assignment for the proposed quorum system. Second, a multichannel quorum-based solution is utilized to avoid collision and overhearing between conflicting wakeup neighbor nodes. According to QMMAC, each node will have a unique channel with each of its next-hop forwarders to communicate through, and hence the throughput is increased, and the end-to-end delay is reduced even further. Simulation results indicate that a great improvement is achieved by QMMMAC in terms of energy efficiency, throughput and end-to-end delay.

## Figures and Tables

**Table 1:** Simulation parameters.

Simulation Parameter	Value
Simulation Time	7200 s
Transmitting power	0.66 W
Receiving power	0.35 W
Sleeping power	0 W
Idle power	0.35 W
Data packet length	40 B
Control packet length	16 B
